# A True Stomachic

**Published:** 1890-03-29

**Authors:** 


### A TRUE STOMACHIC.

This term has been applied to a number of drugs, mostly bitters, as well as to rhubarb in small doses, which possess more or less the power of enhancing the appetite and improving the digestion. But Dr. Penzoldt, of Erlangen, calls attention to a new and artificial body, which, possessing these properties in a pre-eminent degree, seems well to deserve the name that he has given it, of orexin. Its proper chemical designation is phenyldihydrochinazolin, but this body being very insoluble he employs the hydrochlorate. It presents the appearance of colourless crystals, needle shaped and containing two molecules of water of crystalization. The symptoms induced by toxic doses in animals give no clue to its *modus operandi* as a therapeutic agent, though indicating the necessity for caution in its employment. These are dyspnœa, slow respiration, accelerated action of the heart, transient paralyses, vomiting, a darkening of the colour of the blood, with destruction of the red corpuscles. In doses not exceeding 1 gram, it produces in the human subject slight nausea and vertigo, with flushing of the face and a sense of heat and fulness in the head; but these or smaller doses are followed by a surprising increase of appetite. Two medical students, Hofmann and Munter, experimenting on themselves, found that a sense of hunger returned about an hour earlier after a meal than without it; and while taking it in doses of 0.25 grams they could not avoid eating twice as much as before, while the digestion was fully equal to the additional demand. Dr. Penzoldt has avoided it in the more acute forms of gastric catarrh, in which he feared it might act as an irritant, but gives it in ½-gram doses twice or three times a day in anorexia, after operations, in phthisis, pleurisy, anæmia, atonic dyspepsia, and other conditions where it seemed desirable to increase the appetite. He has published the results in 36 cases. In five of these the results were negative, and in five inconsiderable, but in the remaining 26 they were well marked. Many of these were out-patients, and in none were any suggestions or leading questions given, the information being volunteered by the patients, who were surprised at the change in their sensations. Nor was the appetite thus induced of the nature of craving for food; the digestion improved concurrently with the desire for food, and several phthisical patients gained remarkably in weight, and in one case as much as seven pounds in three weeks, and others no less though slower. Sometimes the improvement appeared after the first dose, in others not until a few days. Orexin has been patented by Dr. Paal, its discoverer, and is manufactured by Messrs. Kalle and Co., of Biebrich.

